# Targeted Inhibitory Effect of Lenti-SM22alpha-p27-EGFP Recombinant Lentiviral Vectors on Proliferation of Vascular Smooth Muscle Cells without Compromising Re-Endothelialization in a Rat Carotid Artery Balloon Injury Model

**DOI:** 10.1371/journal.pone.0118826

**Published:** 2015-03-11

**Authors:** Liang Jing, Wenlong Wang, Shuangshuang Zhang, Minjie Xie, Daishi Tian, Xiang Luo, Daowen Wang, Qin Ning, Jiagao Lü, Wei Wang

**Affiliations:** 1 Department of Neurology, Tongji Hospital, Tongji Medical College, Huazhong University of Science and Technology, Wuhan, P.R. China; 2 Department of Cardiovascular Medicine, Tongji Hospital, Tongji Medical College, Huazhong University of Science and Technology, 1095 Jiefang Avenue, Wuhan, 430030, China; 3 Department and Institute of Infectious Disease, Tongji Hospital, Tongji Medical College, Huazhong University of Science and Technology, Wuhan, P.R. China; UNC Eshelman School of Pharmacy, UNITED STATES

## Abstract

**Aims:**

In-stent restenosis remains a serious problem after the implantation of drug-eluting stents, which is attributable to neointima formation and re-endothelialization. Here, we tried to find a new method which aims at selectively inhibiting proliferation of vascular smooth muscle cells (VSMC) proliferation without inhibition of re-endothelialization.

**Methods and Results:**

We used the smooth muscle-specific SM22alpha promoter in a recombinant lentiviral vector to drive overexpression of cell-cycle inhibitor, p27, in VSMCs. p27 effectively inhibited VSMC proliferation mediated by cell cycle arrest at the G0/G1 checkpoint. The SM22alpha-p27 lentiviral vector inhibited VSMC proliferation more effectively than paclitaxel. Rats infected with Lenti-SM22alpha-p27 had a significantly lower intima/media (I/M) ratio and also showed inhibition of restenosis on day 28 after balloon injury. Moreover, the repair of injured endothelium, and re-endothelialization of the carotid artery wall, was not affected by the smooth muscle cell-specific expression of p27.

**Conclusion:**

A recombinant lentiviral vector carrying the SM22alpha promoter was used to effectively infect and selectively overexpress p27 protein in VSMCs, leading to inhibition of intimal hyperplasia without compromising endothelial repair.

## Introduction

Percutaneous transluminal coronary angioplasty has been reported to result in a restenosis rate of about 40% to 50% [1, 2]. The use of bare metal stents decreases the restenosis rate to about 30%, and there is an intense focus on the use of DES (drug-eluting stents) which reduces the rate of early restenosis in patients with coronary artery disease to 10%–30% [3, 4]. The challenges with using DES include: 1) non-selective drug intervention which not only effectively inhibits the proliferation of VSMCs (vascular smooth muscle cells), but also inhibits endothelial proliferation, and the progression of re-endothelialization which can result in a loss of contact inhibition in VSMCs [5]. Although anticancer drugs have been shown to effectively inhibit VSMC proliferation, this is accompanied by a restenosis rate of 5% -10%, and anticancer drugs such as paclitaxel cause hyperlipidemia with bone marrow suppression and side effects [6, 7]. An ideal coating drug should therefore selectively inhibit the proliferation of VSMCs while maintaining the functional integrity of VECs (vascular endothelial cells).

SM22alpha is a 22-kDa protein, which has structural homology to the vertebrate thin filament myofibrillar regulatory protein calponin. SM22alpha is not physically associated with the contractile apparatus and plays an important functional role in smooth muscle cells. The SM22alpha promoter is thought to serve as an excellent model system to examine the molecular mechanisms regulating SMC-specific gene expression [8–10]. Transcription from the SM22alpha promoter occurs in smooth-muscle cells in the absence of factors binding to CC (A/TriCh) 6GG (CArG box) or CANNTG (E box) motifs [11]. The SM22 5’-flanking sequence was shown to be necessary and sufficient to efficiently drive the transcription of a luciferase reporter gene in both primary rat aortic VSMCs and A7r5 cells in vitro [12].

Cell division is tightly regulated by the expression and activation of cyclins, CDKs (cyclin-dependent kinases) and CDKIs (cyclin-dependent kinase inhibitors) [13, 14]. P27^kip1^, a CDKI, belongs to the Cip/Kip family including proteins like p21Cip1/Waf1, p27kip1 and p57Kip2, which interact with a broad range of CDK-cyclin complexes. Endogenous p27^kip1^ has been shown to block cell cycle progression at the G1/S checkpoint [15, 16]. Studies investigating the role of p27^kip1^ in cardiovascular disease showed that patients with the p27kip1-838AA genotype have a decreased risk of in-stent restenosis. This corresponded with enhanced promoter activity of the-838A allele, and may explain decreased smooth muscle cell proliferation in these patients [17].

An ideal therapeutic goal following percutaneous coronary intervention is to inhibit restenosis without affecting the function of endothelial cells. In this study, we designed the Lenti-SM22alpha-p27-EGFP (enhanced green fluorescence protein) lentiviral vector, which contains a VSMC-specific SM22alpha promoter driving the expression of the CDKI p27, along with an EGFP marker. This strategy was used to selectively regulate the proliferation of VSMCs without affecting VECs.

## Materials and Methods

### Construction of Lentiviral vectors

The full-length SM22alpha promoter sequence tagged with p27 (stored in our laboratory) and the EGFP gene were used to construct lentiviral vectors. EGFP alone was used as a control. Recombinant virus was produced at Shanghai Genechem Corporation, China using the ViraPower system (Invitrogen).

### Cell Culture

VSMCs and VECs were prepared from the thoracic aortas of male Wistar rats obtained from the CDC of Hubei province (30 animals, 120–180g). The rats were sacrificed by cervical dislocation. Aortas were dissected from the renal artery to the descending aorta, removed from adventitia, washed in PBS, and dissected away from the endometrial tissue. For VSMCs, aortas were cut into 1 mm^2^ pieces, and incubated for 2 h with digestion buffer (DMEM’s F-12 medium supplemented with 250 U/ml collagenase II) at 37°C. After centrifugation for 5 min at 1000 rpm, the supernatant was discarded, and cells were incubated at 37°C with standard medium (Dulbecco’s modified Eagle’s medium supplemented with 2 mM L-glutamine and 10% fetal bovine serum) in the presence of 5% CO_2_. The purity of VSMCs was checked by immunofluorescence staining of α-smooth muscle actin. For VECs, aortas were inverted gently, ligated at both ends, directly affixed to the pre-coated flasks (rat tail collagen type I for 30 min at 37°C). The cells were incubated with standard medium (M199 medium supplemented with 2 mM L-glutamine, 25 U/ml heparin and 20% fetal bovine serum) at 37°C in the presence of 5% CO_2_. Cells from the initial plating were designated as P0 cells. Cells were trypsinized with 0.25% trypsin-phosphate buffered saline (PBS; Sigma, USA). All experiments were performed with P0 cells at the first (P1) or the second (P2) plating. The purity of VECs was checked by immunofluorescence staining of vWF (von Willebrand factor). The VSMCs and VECs were seeded at the indicated density and cultured for 24 h. Cells were then serum-starved (standard medium, 0.1% fetal bovine serum) for 12 h. For lentivirus infection, 5 μl of PBS (control), Taxol (10 μmol/ml, Sigma), Lenti-SM22alpha-EGFP, or Lenti-SM22alpha-p27-EGFP (4 x 108 TU/ml) were injected into the culture medium, and incubated for 8 h. The remaining solution was removed, then the number of transduced cells was estimated by Flow Cytometric Analysis (see details in supplementary material).

### Immunofluorescence staining

The VSMC and VEC cultures were prepared from 6 rats each. Cells were seeded at a density of 4 × 10^4^/ml on coverslips in 6 well plates which were pre-incubated with 0.1 mg/ml polylysine in PBS for 30 min at 37°C. The coverslips were removed at the pre-designed time points with sterile tweezers, washed with sterile PBS three times, fixed with methanol for 15 min, and incubated with antibody blocking solution for 8 hours at 4°C. The coverslips were then incubated with 1:1000 dilution of rabbit anti-p27, or a 1:500 dilution of goat anti-EGFP (all from Abcam) for 8 hours at 4°C. After that, the coverslips were incubated with a 1:1000 dilution of Cy3-linked anti-rabbit antibody, or a 1:1000 dilution of FITC linked anti-goat antibody (all from Jackson) for 1 hour at 37°C, and then incubated with DAPI (0.5 μg/ml, Sigma) for 5 min at 37°C. The coverslips were visualized with an Olympus, BX51-type fluorescence microscope. Each intervention group was done in triplicate.

### Flow Cytometric Analysis

The VSMC and VEC cultures for flow cytometric analyses were prepared from 6 rats each. Cells were plated at a density of 4 × 10^4^/ml in 6 cm dishes. Cells were trypsinized after 72 h, fixed with ice-cold 75% ethanol, and incubated with 50 μg/ml PI for 2 h at 4°C. The cells (10,000) were analyzed by flow cytometry on a FACSort (BD Biosciences, San Jose, CA). Each intervention group was done in triplicate.

### Western Blot Analysis

The VSMC and VEC cultures for Western blot analyses were prepared from 3 rats each. Cells were plated at a density of 4 × 10^4^/ml in 10 cm dishes. After 72 hours, cells were washed with ice-cold PBS, incubated with RIPA lysis buffer for 30 min at 4°C and centrifuged for 25 min at 12,000 rpm at 4°C. Supernatants were mixed with 5xSDS sample buffer and heated to 110°C for 15 min. Protein concentrations were determined using Rotiquant reagent (Carl Roth). Standard gel electrophoresis and blotting techniques were used (Bio-Rad). Primary antibodies were diluted 1:1000, and secondary antibodies were diluted 1:10,000. Bands were analyzed with an enhanced chemiluminescence protocol and exposed to X-ray film (Kodak Company, USA). The developed blots were analyzed using the Gel Imaging Analysis system (Gene Genius Company, USA).

### Rat Carotid Artery Balloon Injury

All surgical procedures and postoperative care were performed in accordance with guidelines of US National Institutes of Health, and the Animal Care and Use Committee of Huazhong University of Science and Technology. Protocols were approved by the Ethics Review Board of Tongji Medical College. Male Wistar rats (36 animals, 350 to 450 g) obtained from the CDC of Hubei province, were anesthetized with 6% chloral hydrate (300 mg/kg intraperitoneally). All animals received an infusion of heparin (100 U/kg) before the operation. The left common carotid artery was injured with a Fogarty 2F catheter (Baxter Healthcare, USA), and the catheter was advanced into the left common carotid artery from the incision on the external carotid artery. The balloon was inflated to 5 atmospheres for 30s, then the balloon catheter was pulled back and then advanced 3 times. Rats in sham group underwent the same operation, except that the balloon was not inserted. For lentivirus infection, after the balloon injury, the balloon catheter was removed. Then 50 μl of PBS (control), Lenti-SM22alpha-EGFP, or Lenti-SM22alpha-p27-EGFP (2x10^9^ TU/ml) were infused into the blood vessel, and incubated for 30 minutes. The remaining solution was removed, then the external carotid artery was ligated and the skin incision was closed. All animals were sent back to the SPF animal experimental center of Tongji Medical College of Huazhong University of Science and Technology.

### Histological analysis and morphometry

Rats were sacrificed by cervical dislocation after 7 days, 14 days or 28 days after surgery. The injured segments of the artery were collected and fixed in 4% paraformaldehyde for 24 hours. Then all the samples were embedded in paraffin and transverse histological sections (4 μm) were made from each segment and stained with hematoxylin-eosin. Morphometry was performed using a video microscope (Olympus DP72). The area of the neointima, the area of the lumen and the ratio of the areas of neointima to media (I/M) were calculated using ImageJ2x-2.1.4.6 software (NIH, USA). Five discontinuous sections from each vessel were measured in a rat, and three rats were used in each experimental group.

### Statistical analysis

Results were expressed as means±SEM. Statistical significance between two groups was analyzed by Student’s t-test. Comparison among multiple groups was made using one-way ANOVA. A P value <0.05 was considered statistically significant.

## Results

### Selective efficiency of SM22alpha promoter in VSMC

**Fig 1:**
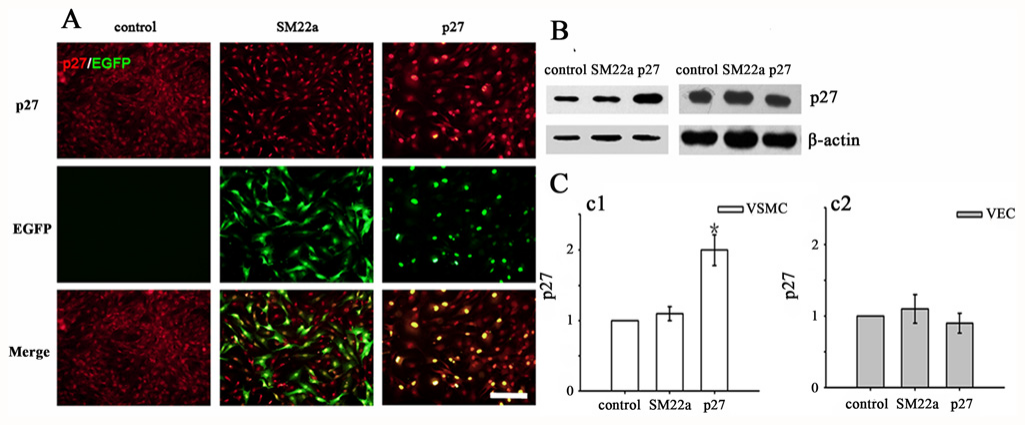
Vascular smooth muscle cells (VSMCs) overexpressed p27 under the control of the SM22alpha promoter in vitro. A. Immunofluorescence double staining of cells in the control, SM22a and p27 VSMC groups. P27 stained red while EGFP stained green. The images represent nuclear overexpression of p27 and EGFP in the p27 control group and SM22a group. Scale bar: 100 μm. B. Western blot analysis to evaluate p27 expression in VSMCs (b1) and VECs (b2). C. Western blot analysis of p27 content in VSMCs (c1) and VECs (c2). *The p27 content of Lenti-SM22alpha-p27 infected VSMCs was higher compared to control and SM22a groups. Error bars represent the mean ± S.E. (n = 3).

VSMCs and VECs were infected with Lenti-SM22alpha-p27-EGFP or Lenti-SM22alpha-null-EGFP at an multiplicity of infection of 10 and harvested after 72 h. The infection efficacy of vectors was analyzed using fluorescence emission of EGFP by FACS (S1 Fig.). In VSMCs, Lenti-SM22alpha-EGFP and Lenti-SM22alpha-p27-EGFP vectors induced significantly higher expression of EGFP compared to control group (S1 Fig., Lenti-SM22alpha-EGFP group 95.45 ± 3.17 vs. 0.21±0.01, p <0.01, Lenti-SM22alpha-p27-EGFP group 90.39±7.24 vs. 0.21±0.01, p <0.01). But there was no significant difference of infection efficacy between two vector groups (S1 Fig.). Expression levels of p27 and EGFP were evaluated in the VSMC groups using immunofluorescence staining (Fig. 1 a1–a3). P27 was overexpressed in the nucleus in the Lenti-SM22alpha-p27 group (Fig. 1 a3), whereas p27 in the Lenti-SM22alpha-null group was distributed in the nucleus along with a certain amount of cytoplasmic expression (Fig. 1 a2). EGFP expression was not detected in the control group (Fig. 1 a4), which was distributed in the cytoplasm in the Lenti-SM22alpha-null group (Fig. 1 a5), and was aggregated in the nucleus in the Lenti-Sm22alpha-p27 group (Fig. 1 a6). There was a significant nuclear expression of p27 and EGFP expression in the Lenti-SM22alpha-p27 group (Fig. 1 a9).The Lenti-SM22alpha-p27 VSMC group showed a significantly higher expression of p27 protein compared to the Lenti-SM22alpha-null group (Fig. 1 b1, 2.154±0.223 vs. 1.082±0.143, p <0.01). However, there was no statistically significant difference in p27 overexpression among the three VEC groups (Fig. 1 b2). These data were consistent with the data from Western blot analyses (Fig. 1 c1, and c2).

### Influence of Lenti-SM22alpha-p27 on VSMC Proliferation

**Fig 2:**
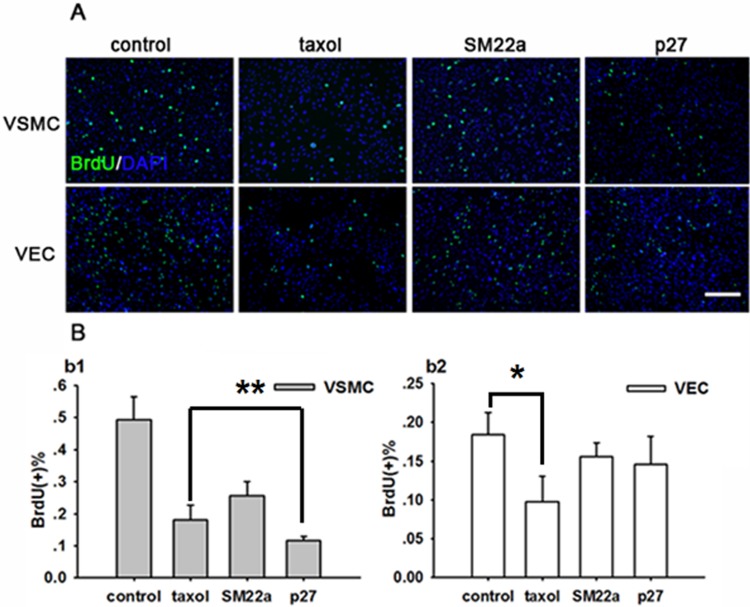
Targeted inhibition of VSMC proliferation in vitro by Lenti-SM22alpha-p27 vectors compared with paclitaxel. A. BrDU immunofluorescence staining of BrdU in VSMCs and VECs (control, taxol, SM22a and p27 groups). BrdU is shown in green and DAPI is shown in blue. Scale bar: 200 μm. B. BrdU positive cells in VSMCs (b1) and VECs (b2). *(p<0.05, taxol vs. control). **(p<0.05, taxol vs. p27). Error bars represent the mean ± S.E. (n = 3).

BrdU staining data showed that VSMCs treated with taxol or recombinant lentivirus, especially Lenti-SM22alpha-p27, had a significantly lower density compared with the sham group. However, the density of VECs was significantly decreased only in the taxol group. In the VSMC group, Lenti-SM22alpha-p27 inhibited the proliferation of VSMCs more effectively than paclitaxel (Fig. 2 b1, taxol group 0.493±0.072 vs. 0.180±0.046, p<0.01, Lenti-SM22alpha-p27 group 0.493±0.072 vs. 0.117±0.013, p<0.05). Lenti-SM22alpha-null did not significantly inhibit VSMC proliferation. In the VEC group, paclitaxel significantly inhibited VEC proliferation compared to control cells (Fig. 2 b2, taxol group 0.184±0.028 vs. 0.097±0.033, p<0.05). In contrast, Lenti-SM22alpha-null and Lenti-SM22alpha-p27 did not significantly inhibit the proliferation of VECs.

### Effect of Lenti-SM22alpha-p27 on the Cell Cycle of VSMCs

**Fig 3:**
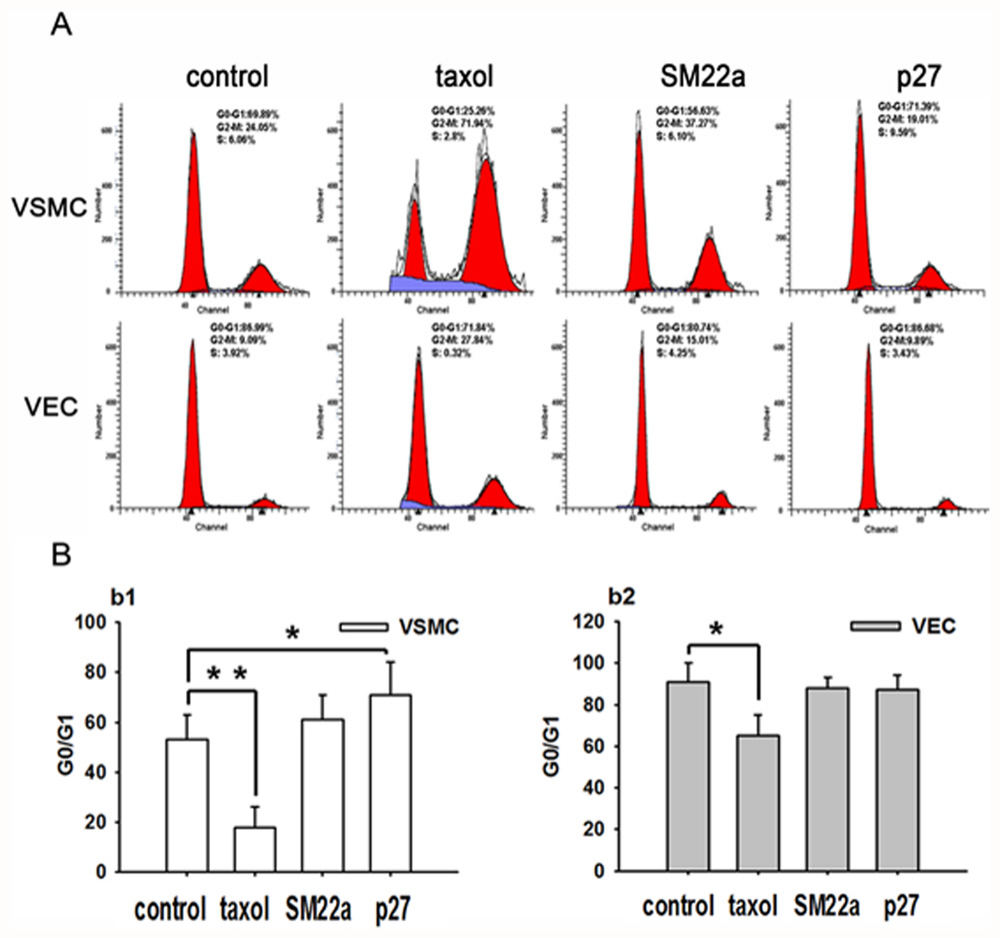
G0/G1 arrest in VSMCs (Lenti-SM22alpha-p27 group compared with paclitaxel group) in vitro. A. FACS data for VSMCs and VECs (control, taxol, SM22a, and p27 groups). B. G0/G1 distribution in VSMCs (b1) and VECs (b2). *(p<0.05, taxol vs. control). **(p<0.05, p27 vs. control). Error bars represent the mean ± S.E. (n = 3).

Taxol induced G2/M cell cycle arrest in VSMCs accompanied by a decrease in G0/G1 phase distribution, while Lenti-SM22alpha-p27 effectively induced G0/G1 arrest (Fig. 3 b1, Lenti-SM22-alpha-p27 group 71.827±13.547 vs. control group 53.741±11.233, p<0.05). In VECs, taxol effectively induced G2/M cell cycle arrest accompanied by a decrease in G0/G1 phase distribution. Lenti-SM22alpha-p27 did not significantly affect the cell cycle in VECs (Fig. 3 b2).

### Effect of Lenti-SM22alpha-p27 in Rat Carotid Artery Balloon Injury model

**Fig 4:**
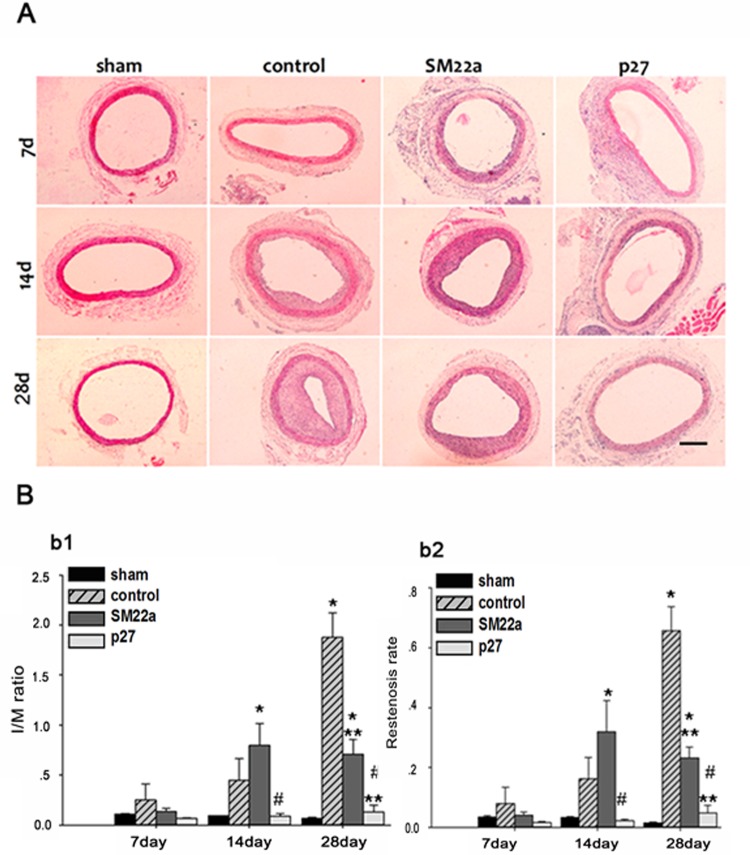
Targeted effect of Lenti-SM22alpha-p27 vectors in Rat Carotid Artery Balloon Injury model. A. Histological analysis of sham, control, SM22a and p27 carotid artery groups at 7, 14, and 28 days after the balloon injury. Bar: 500 μm. B. Intima/Media ratio (b1) and Restenosis ratio (b2) of different groups. *(p<0.05, 14 days SM22a vs. sham, 28 days control vs. sham and SM22a vs. sham). **(p<0.05, 28 days SM22a vs. control, p27 vs. control). #(p<0.05, 14 days p27 vs. SM22a, 28 days p27 vs. SM22a). Error bars represent the mean ± S.E. (n = 3).

At twenty-eight days after balloon injury, histological analysis (Fig. 4A) showed a significant degree of neointimal hyperplasia in the injured artery which was exposed to PBS or Lenti-SM22alpha-null compared with the sham group. The I/M ratio was significantly lower in the group infected with Lenti-SM22alpha-p27 compared to the groups treated with PBS (0.129±0.072 vs. 1.878±0.243, p<0.01), or Lenti-SM22alpha-null (0.129±0.072 vs. 0.708±0.148, p<0.01) (Fig. 4B). The group infected with Lenti-SM22alpha-p27 also had a significantly lower restenosis rate compared to the groups treated with PBS (0.048±0.025 vs. 0.657±0.081, p<0.01), or Lenti-SM22alpha-null (0.0487±0.025 vs. 0.233±0.035, p<0.01) (Fig. 4B), which indicated the development of neointimal hyperplasia.

### Lentivirus-SM22alpha-p27-EGFP inhibits neointima and media VSMCs proliferation

**Fig 5:**
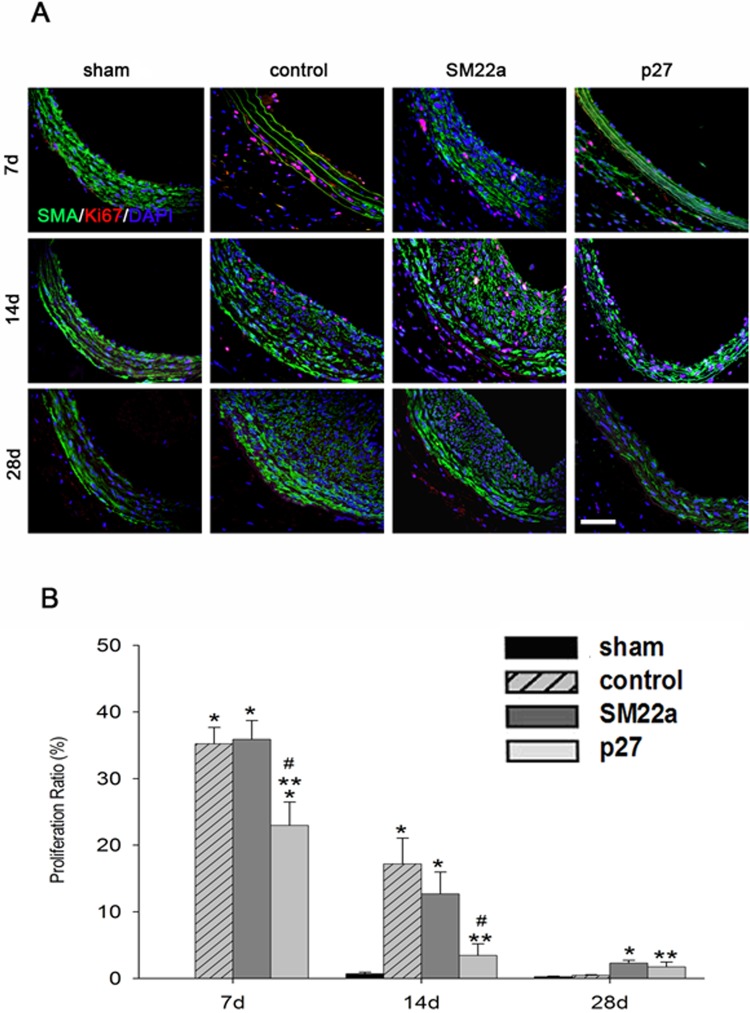
Inhibition of neointimal hyperplasia with Lenti-SM22alpha-P27 lentivirus vector in Rat Carotid Artery Balloon Injury models. A. Ki67 immunofluorescence staining in sham, control, SM22a and p27 carotid artery groups at 7, 14, and 28 days after the balloon injury. Ki67 is shown in red, SMA is shown in green while DAPI is shown in blue. Scale bar: 300 μm. B. Measurement of proliferation ratio in different groups using Ki67 staining. *(p<0.05, 7 days control, SM22a, p27 vs. sham. 14 days control, SM22a vs. sham, 28 days SM22a vs. sham). **(p<0.05, p27 vs. control in 7 days, 14 days and 28 days). #(p<0.05, p27 vs. SM22a in 7 days, 14 days and 28 days). Error bars represent the mean ± S.E. (n = 3).

Neointimal and medial VSMC proliferation was assessed by staining of SMA (α-smooth muscle actin), ki67 and DAPI in-situ (Fig. 5A). The Lenti-SM22alpha-p27 group displayed a significant inhibition of proliferation compared to the control group (day 7, 22.96±3.51 vs. 35.23±2.45, p<0.01; day 14, 3.46±1.74 vs. 17.19±3.89, p<0.01). In contrast, the Lenti-SM22alpha-null group did not significantly inhibit VSMC proliferation (day 7, 35.89±2.84 vs. 35.23±2.45, p = 0.855; day 14, 12.69±3.27 vs. 17.19±3.89, p = 0.214). These results suggest that overexpressed p27 played a role in suppression of VSMC growth.

### Lentivirus-SM22alpha-p27-EGFP does not suppress re-endothelialization of injured arteries

**Fig 6:**
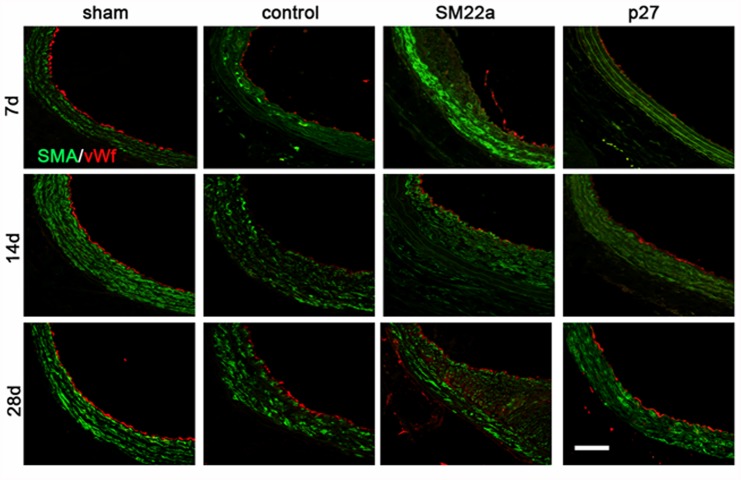
Re-endothelialization with Lenti-SM22alpha-p27 in Rat Carotid Artery Balloon Injury models. vWF immunofluorescence staining in sham, control, SM22a and p27 carotid artery groups at 7, 14, and 28 days after the balloon injury. vWF is shown in red, SMA is shown in green. Scale bar: 300 μm.

Compared with the sham group, the endothelium, internal elastic and part of media were damaged after balloon injury. The expression of vWF, an endothelial marker, was evaluated by immunofluorescence on days 7, 14 and 28 after injury. Overexpression of p27 did not affect re-endothelialization compared to the control group (Fig. 6).

## Discussion

Based on the finding that neointimal formation is the major cause of ISR (in-stent restenosis), a number of strategies have been developed to inhibit neointimal formation in cells and in animal models [18–21]. However, these have proved unsuccessful in clinical trials. Although DES showed early promise in effectively inhibiting ISR, current studies show a 10%~30% ISR rate accompanied by LSR after implantation of DES. The DES strategy has been shown to inhibit neointimal formation as well as re-endothelialization underscoring the fact that the ideal therapeutic strategy should aim for a selective antiproliferative effect on VSMCs without targeting VECs [22]. In this study, we prepared a recombinant lentiviral vector carrying the SM22alpha promoter to selectively overexpress p27 protein in VSMCs, leading to inhibition of intimal hyperplasia without compromising endothelial repair.

The cell cycle mechanisms underlying the inhibition of proliferation of VSMCs and VECs after DES implantation remain unclear. However, the G1/S transition point has been shown to play an important role in VSMCs under conditions of mechanical or inflammatory injury [23]. P27^kip1^ regulates the G1/S transition by inhibiting the activity of cyclin E/CDK2, which is considered to be an endogenous regulator in VSMCs. The p27^kip1^ levels are regulated by drugs such as rapamycin, sirolimus and paclitaxel [24–26], and paclitaxel-eluting stents were demonstrated to substantially reduce early restenosis in clinical trials. Although paclitaxel has been traditionally considered to be an inhibitor of the G2/M transition point [27, 28], recent reports have shown that paclitaxel inhibited cell cycle progression by inducing a G0/G1 cell cycle arrest. Based on these data, we used a lentiviral vector overexpressing p27 under the control of the SM22alpha promoter, and showed that p27-mediated G0/G1 cell cycle arrest played a role in inhibition of proliferation of VSMCs.

Re-endothelialization is a vascular remodeling process which may be affected by mechanical or inflammatory injury [29]. Re-endothelialization was shown to balance neointimal hyperplasia and matrix accumulation in the early phase of artery remodeling [22]. In the late phase of artery remolding, upregulation of p27 in VECs may inhibit occurrence of late stent thrombosis [30]. Stent implantation and the potent anti-mitotic effect of eluting drugs may impair the physiological process of endothelial regeneration, leading to prolonged vascular healing and late thrombosis. Direct inhibition of proliferation of VECs and VSMCs by paclitaxel could lead to late stent thrombosis and ISR [31]. In contrast, overexpression of p27 using the Lenti-SM22alpha-p27 vector selectively inhibited the proliferation of VSMCs without affecting VECs, suggesting the benefits of using the recombinant lentivirus strategy to inhibit ISR.

We showed that the p27-overexpressing recombinant lentivirus specifically infected VSMCs and efficiently inhibited proliferation. A number of recent studies have investigated techniques to improve DES [20, 32–37]. Some studies focused on identifying novel chemotherapeutic drugs which can regulate the cell cycle by targeting specific pathways in lesion sites [38, 39], while others aimed at evaluating VSMC-specific factors such as vascular endothelial growth factor and C-type natriuretic peptide [22, 40–42]. The application of these agents in a clinical setting is limited by factors such as their non-selective inhibitory effect on VSMCs, plasma concentrations and long-term sustainable release of the relevant agents. In contrast, gene therapy not only specifically targets and inhibits VSMCs proliferation but also achieves long-term expression at localized sites. The possibility of using recombinant lentiviral vectors for clinical applications is further supported by the demonstration of the clinical applications of recombinant adenovirus therapy [43–45].

In this study, we used carotid intrathecal injection as a delivery method [46–48], since this has been shown to inhibit the entry of the recombinant lentivirus into the circulation and assure viral content in all layers of the vessel. Compared with the sham group, the endothelium, internal elastic and part of the media were damaged after balloon injury in vivo. A significant degree of neointimal hyperplasia was observed at 28 days after injury, compared to the 7 days and 14 days groups. The delivery of Lenti-SM22alpha-p27 significantly decreased the development of neointimal hyperplasia. vWF staining showed that delivery of p27 did not significantly affect re-endothelialization in this group compared with the other groups. Endothelial repair was almost complete at 28 days. Based on these data, we suggest that this recombinant lentivirus strategy safely and effectively inhibited neointima formation in a rat balloon injury model.

In conclusion, we demonstrated that overexpression of p27 by Lenti-SM22alpha-p27 safely and efficiently inhibited VSMC proliferation in vitro and in vivo, without affecting VECs. Our further investigations aim at the construction of recombinant lentivirus-coated stents and testing such agents with DES.

## Supporting Information

S1 Fig Infection efficacy of SM22a promoter in vitro. A: FACS data for VSMCs (control, SM22a and p27 groups). B: EGFP positive cell ratio in VSMCs. *(p<0.01, SM22a vs. control). **(p<0.01, p27 vs. control). Error bars represent the mean ± S.E. (n = 3).
(DOC)
