# Supplementary material for: Targeted Inhibitory Effect of Lenti-SM22alpha-p27-EGFP Recombinant Lentiviral Vectors on Proliferation of Vascular Smooth Muscle Cells without Compromising Re-Endothelialization in a Rat Carotid Artery Balloon Injury Model
Source: PLoS One. 2015 Mar 11;10(3):e0118826. doi: 10.1371/journal.pone.0118826 (PMC4356572; doi:10.1371/journal.pone.0118826)
Supplement: S1 Fig — A: FACS data for VSMCs (control, SM22a and p27 groups). B: EGFP positive cell ratio in VSMCs. *(p<0.01, SM22a vs. control). **(p<0.01, p27 vs. control). Error bars represent the mean ± S.E. (n = 3). (DOC) [file pone.0118826.s001.doc]

**Supplementary information**

**Targeted inhibitory effect of Lenti-SM22alpha-p27-EGFP recombinant lentiviral vectors on proliferation of vascular smooth muscle cells without compromising re-endothelialization in a rat carotid artery balloon injury model**

Liang Jing#^1^, Wenlong Wang#^2^, Shuangshuang Zhang^2^, Minjie Xie^1^, Daishi Tian^1^, Xiang Luo^1^, Daowen Wang^2^, Qin Ning^3^ , Jiagao Lü^2^, Wei Wang^1^

^1^Department of Neurology, Tongji Hospital, Tongji Medical College, Huazhong University of Science and Technology, Wuhan, P.R. China

^2^ Department of Cardiovascular Medicine, Tongji Hospital, Tongji Medical College, Huazhong University of Science and Technology, 1095 Jiefang Avenue, Wuhan, 430030, China

^3^ Department and Institute of Infectious Disease, Tongji Hospital, Tongji Medical College, Huazhong University of Science and Technology, Wuhan, P.R. China

**#** The authors contributed equally to this work.

Total word count: 5242

*Correspondence should be addressed to Dr. W.Wang. ([wwang@vip.126.com](mailto:wwang@vip.126.com)) or **L**ü J (lujiagao[@tjh.tjmu.edu.cn](mailto:bubitao@tjh.tjmu.edu.cn)), Department of Neurology, Tongji Hospital, Tongji Medical College, Huazhong University of Science and Technology, Wuhan 430030, P.R.China,Tel: +86-27-83663657,Fax: +86-27-83663657.

**Methods**

Flow Cytometric Analysis to determine the infection efficacy in vitro

VSMC cultures for flow cytometric analyses were prepared from 6 rats each. Cells were plated at a density of 4 × 10^4^/ml in 6 cm dishes. For lentivirus infection, 5 µl of PBS (control), Lenti-SM22alpha-EGFP, or Lenti-SM22alpha-p27-EGFP (4 x 10^8^ TU/ml) were injected into the culture medium, and incubated for 8 h. Cells were trypsinized after 72 h, centrifuged for 5min at 800rpm. The supernatant was discarded and cells were collected with sterile PBS at 4°C. Cells were analyzed by flow cytometry on a FACSort (BD Biosciences, San Jose, CA). Each intervention group was done in triplicate.

**Figure S1.** Infection efficacy of SM22a promoter in vitro. A: FACS data for VSMCs (control, SM22a and p27 groups). B: EGFP positive cell ratio in VSMCs. *(p<0.01, SM22a vs. control). **(p<0.01, p27 vs. control). Error bars represent the mean ± S.E. (n = 3).


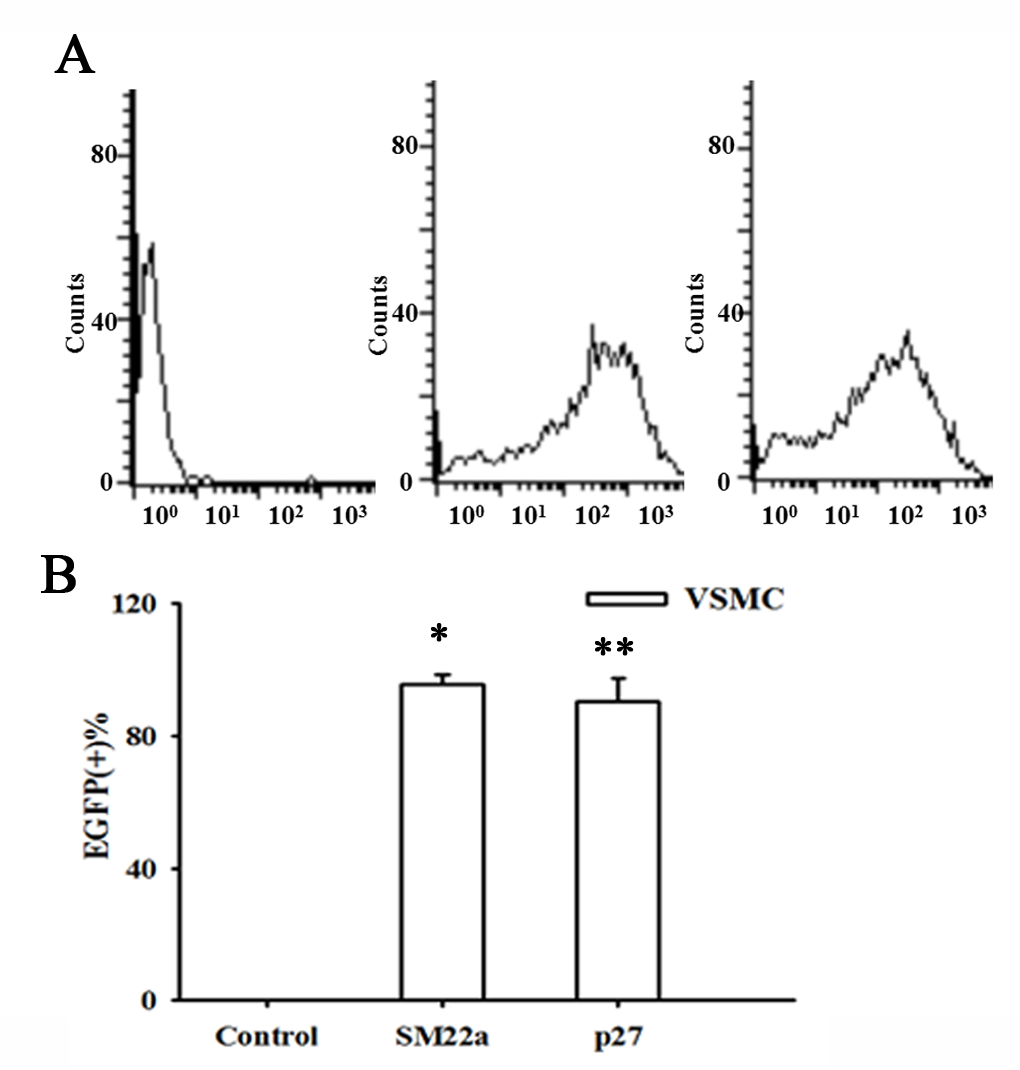


**Figure S1**
